# "What lies beneath it all?" – an interview study of GPs' attitudes to the use of guidelines

**DOI:** 10.1186/1472-6963-8-218

**Published:** 2008-10-22

**Authors:** Benedicte Carlsen, Ole F Norheim

**Affiliations:** 1 Stein Rokkan Centre for Social Studies, The University of Bergen, Nygårdsgaten 5, 5015 Bergen, Norway; 2 Department of Public Health and Primary Health Care, The University of Bergen, Kalfarveien 31, 5018 Bergen, Norway

## Abstract

**Background:**

General practitioners (GPs) adopt clinical practice guidelines to varying degrees. Several factors have been found to influence application of guidelines in practice and the GP is apparently the key actor. Studies are needed to increase our understanding of how GPs' attitudes influence their use of guidelines. In this study we explored GPs' attitudes to guidelines.

**Methods:**

In 2007 we conducted six semi-structured group interviews with a purposive sample of 27 Norwegian GPs. The participants were encouraged to discuss guidelines they were familiar with, the evidence base of guidelines, professional autonomy and doctor-patient relations. We used thematic content analysis to extract central themes and arguments.

**Results:**

When deciding whether tfollow guideline recommendations, GPs consider whether guidelines are trustworthy, whether they suit patients and whether the recommended action is feasible. There were two important findings. First, the GP's were concerned that guidelines may be more heavily influenced by economic considerations than clinical ones. Second, in contrast to earlier findings, changes in recommendations and disagreement between experts were mostly viewed positively.

**Conclusion:**

This study underscores the need for transparency in the process of development and implementation of guidelines. To enhance the use of guidelines, primary care physicians should be involved in the process of developing guidelines and the process should be transparent and explicit regarding the evidence base and economic considerations.

## Background

Clinical guidelines can be thought of as "generic decisions – recommendations intended for a collection of patients rather than for a single patient" [1]. Clinical practice guidelines have also been defined as "user-friendly statements" for a collection of patients, based on the best external evidence [2]. Although the demarcation is not always clear, it is useful to distinguish between proscriptive (regulatory) guidelines which are associated with rationing health care, and prescriptive guidelines which aim to encourage best clinical practice even if this recommends increased use of interventions [3]. Descriptive studies indicate that clinical practice guidelines are read and adopted to varying degrees [4,5]. In particular it seems to be difficult to secure adherence to guidelines among general practitioners (GPs), who are in many ways an autonomous group [6,7]. A recent Norwegian survey of 850 GPs concluded that only a few of the current guidelines are known to and used by GPs [8].

Surveys on the implementation of guidelines have categorised some of the major barriers to guideline adherence which can be related to practitioner, patient, organisational or guideline factors [9-11], although the practitioner appears to be the key actor. There has been a call for explorative studies to increase our understanding of how GPs' attitudes are related to the use of guidelines in medical decision making [12,13], but hitherto only a few studies have explored what lies behind GPs' views of guidelines [3].

A recent review and synthesis of interview studies of GPs' attitudes to guidelines identified common barriers to implementation [3]. This study found that barriers are similar across studies and countries and the barriers described in the qualitative explorative studies do not differ much from barriers identified in quantitative surveys, although the qualitative studies offer a more in-depth understanding of what lies behind GP's attitudes and how they are related. The review study identified six main categories of barriers to the implementation of guidelines: questioning the content of the guideline, applying evidence in practice, preserving a good doctor-patient relationship, professional responsibility, practical issues, and the format of guidelines. The study also showed the usefulness of distinguishing between proscriptive and prescriptive guidelines.

Most qualitative studies have explored clinicians' attitudes to the implementation of just one or a few specific guidelines, while some recent studies include several guidelines for comparison [6,14,15]. These studies investigate the clinician's point of view, but the results are also inevitably coloured by the guidelines in question, as well as the process of implementation and the policy context of implementation. In a more general study, where the participants choose which guidelines to discuss, it is possible to see which guidelines the GPs are currently aware of and which are debated. Carlsen et al's review found only one study of GPs' attitudes to guidelines in general [12], and two that studied different guidelines from a common source (National Institute for Health and Clinical Excellence – NICE) [15,16]. An important barrier identified in these studies is the belief among GPs that guidelines based on research conducted in other settings is not valid in their own general practice. Generally GPs held the opinion that clinical experience and judgement should overrule guidelines. Practical constraints such as lack of time to acquaint oneself with guidelines and the evidence behind them were also central.

The aim of our study was to explore GPs' attitudes to clinical practice guidelines in general. In this paper, we focus on the factors Norwegian GPs emphasize when they consider whether or not to follow guidelines.

### The Norwegian Setting

In a European perspective, Norwegian GPs are presented with relatively few guidelines, and Norwegian general practice is generally characterised by low levels of monitoring and control. In Norway there is yet no superior source of national guidelines for general practice such as NICE is for British GPs. Several government led institutions, as well as some independent and private organisations, and pharmaceutical companies, have all produced clinical guidelines for general practice in Norway in recent years. Comparatively few guidelines are issued by the government led institutions.

## Methods

One of the researchers (the social scientist – BC) conducted six semi-structured group interviews with a purposive sample of 27 Norwegian GPs from the counties of Hordaland (20 GPs) and Oslo (7 GPs). A letter of invitation was sent to 93 doctors' educational groups. The final sample was selected from 11 groups that contacted the research team following the invitation. The sample was chosen to reflect variation in age, gender, professional experience, list size, urbanisation, and patient populations of different socioeconomic levels. The participants in the groups knew each other from earlier group meetings, and the interviews were conducted at the usual place and time for group meetings; usually in the lunch room of one of the participants' practice. At the start of the interviews all participants were informed about anonymity issues and their right to withdraw from the study at any point. Subsequently they signed an informed consent form and filled in a short questionnaire of background information (Table 1).

**Table 1:** Sample profile compared to all Norwegian GPs

**Variable**	Sample*	GPs in Norway**
Number of GPs	27	3862

Male GPs	67%	68%

Age (mean)	45	47

Current list size (mean)	1110	1196

GPs with open list	31%	46%

* Our data from the questionnaire. The proportions given are calculated on the basis of the total number of answers to each question.

** Data from the Norwegian Labour and Welfare Organisation and the Norwegian Medical Association: Available at  and  Accessed August, 2007.

New interviews were initiated until a point of data saturation was reached. Interviews lasted approximately one hour and were recorded and transcribed verbatim.

The interview guide included the following themes:

• What characterises good guidelines?

• Trust in the evidence and the guidelines

• Barriers to using guidelines

• How guidelines influence professional autonomy and relationship with patients

The participants were encouraged to mention specific guidelines and to illustrate their points with examples from practice. However, no definition of clinical practice guidelines was presented by the researchers.

Thematic content analysis [17] was applied to search the data for common themes and arguments. Both authors read the interviews thoroughly and then defined and discussed emergent themes and possible codes until agreement was reached. The first author coded the interviews, while the second author checked and revised the coded texts. Informant validation was conducted by inviting all participants to assess the preliminary manuscript of this paper. The manuscript was distributed to all of the groups, but only one group provided comments.

### Ethical approval

The project has been approved by the Privacy Ombudsman against the privacy and license requirements of the Data Inspectorate in relation to the Personal Data Act and Health Register Act.

## Results

The sample profile was similar to the population of GPs in Norway in terms of age, gender and list size, while the proportion with open lists (i.e., doctors with fewer patients on their list than they would like) was somewhat smaller in our sample (Table 1).

As it was left to the participants to describe guidelines, they referred to a broad spectrum of rules, regulations, recommendations and information which they associated with the concept of guidelines. A limited number of guidelines and related health issues were repeatedly discussed in the groups. The most frequently mentioned were guidelines for: primary prevention of cardiovascular disease, diabetes, antenatal care, COPD/asthma, and mammography screening.

The participants' knowledge of and reported use of guidelines varied, but there was general agreement about which factors are important when considering whether to follow recommendations. The interviews indicate three main categories of factors that influence GPs' decisions about following guidelines (See Table 2 for an overview).

**Table 2:** Questions considered by GPs when deciding whether to follow a guideline

1. Can I trust the recommendations?	a. Are the recommendations driven by economic motives (rationing or profit)?
	b. Is the source trustworthy?

	c. Is the evidence trustworthy?

2. Are the recommendations consistent with this patient's needs?	a. Is it possible to say no to patients' requests without disappointing them?

	b. Are patients likely to comply with recommended treatment?

3. Is following the recommendations feasible?	a. Are the recommendations easily accessible and comprehensible to me and the patient?

	b. Have I got the time to assess and follow the recommendations?

	c. Have I got the necessary skills and resources to follow the recommendations?

### Factors influencing guideline adherence

#### 1. Can I trust the recommendations?

A common predicament for the GPs when assessing guidelines was a lack of trust in the recommendations. The most evident source of distrust among the participants was the concern that economic motives may overshadow clinical considerations. The participants feared that government guidelines designed for cost control and rationing are clinically unsound and do not recommend best practice or adequate health care. It was suspected that hidden economic motives often lie behind guidelines. However an explicit rationing motive was regarded as acceptable.

*I think this is moving the focus away from the patient and towards a set of rules which is not about patients but about money*.

(Male GP, Oslo)

*GP: I am concerned that money is all too important. I am afraid the economic issues will overshadow other important values*.

Researcher: That economic issues are included in the decision without your being aware of it?

*GP: Yes, that that is what lies beneath it all*.

(Female GP, Oslo)

*I think it's reasonable from the authorities' side to have guidelines which say we have economic boundaries, which mean we can't do everything we could, but then it should be stated that that is what is happening. And there aren't many people who dare to say that. So I am worried that guidelines come out with an underlying agenda which we don't get to see*.

(Male GP, Oslo)

Some of the participants found it difficult to "sell" cost-cutting guidelines to patients, while others said that, in the end, patients would understand and follow doctors' advice if they were explicitly informed about cost issues. Both these points of view suggest that the doctor's own attitude to guidelines often is transmitted to the doctor-patient relationship. The doctor's own suspicion of a hidden agenda of cost control was seen as a problem when the doctor had to negotiate new routines with patients, whereas if the doctor had accepted and understood the guidelines, this also seemed to be reflected in patients' attitudes.

*But it is worse with medicines for blood pressure, and then sometimes I think it's a hassle to have to start everyone on thiazides. And then I'm maybe a bit biased so that patients notice. So they get the side effects they expect so we can put them on the other drugs*.

(Female GP, Oslo)

*I see that people think it seems quite reasonable. If the drugs are just as good, and I say we have been told to use it to save a couple of million kroner a year. Then they say, yes, that's fine*.

(Male GP, Hordaland)

Sometimes GPs agreed with health authorities that rationing is necessary and that economic considerations should, therefore, be part of the guideline basis. Nevertheless they had little faith in the economic evaluations behind specific guidelines. They were suspicious that the economic evaluations had been too simplistic, i.e. not including any extras that the implementation of the guideline would entail, or they were doubtful that the health authorities could be trusted to use savings in other more important areas.

*The problem with this type of guidance from the state medicines agency is that nobody counts the extra doctor time used. How much extra treatment costs does the welfare office get for appointments and fees for doctors using time on this? It may not be very much, but it will outweigh a good part of the effect, and we can't see that health economists have looked at this*.

(Male GP, Hordaland)

*It's easy to see the importance of using the right medicines, but it's clear that part of the problem with, for example, reimbursement of drugs is that it appears very random and unfair. When the state medicine's agency says no, the industry goes to the parliament (...) and gets them to speak up for a medicine with little effect. It obviously seems a bit dumb that I'm supposed to sit here in my practice and change all my patients to a cheaper copy of the drug for the state to save money which it then doles out at random to huge pharmaceutical companies*.

(Male GP, Hordaland)

Fear of economic motives led to distrust of both governmental and pharmaceutical guidelines albeit for very different reasons. The GPs frequently referred to the conventional truth that recommendations and courses sponsored by the industry are driven by a chase for profit and cannot be trusted. Some even said that they refused to read such recommendations.

*They both have their agenda. The pharmaceutical industry is for profit and the state wants to save money. So in a way it's two sides of a money issue*.

(Male GP, Hordaland)

*If the pharmaceutical company has financed it, it goes in the bin unread. Even if it might be very good. And much the same applies to certain guidelines from the state. Because the state medicine's agency has a one-sided focus on saving money. You can't always trust what comes from there*.

(Male GP, Hordaland)

In contrast to the scepticism towards proscriptive (regulatory) guidelines from health authorities or the prescriptive (innovative) guidelines from the pharmaceutical industry, guidelines developed by general practitioners were unanimously trusted. The guidelines GPs claimed most adherence to were treatment programmes which were developed by general practitioners or by a multi-disciplinary group that included general practitioners. This was seen both as a guarantee that the guidelines were not economically motivated and also that the authors were familiar with the complex reality of general practice.

*I feel that the further they are from us, from knowing what daily life is like for us, the less faith I have in them. I have most faith in what is presented by those who know what our every day is like*.

(Female GP, Oslo)

*Clearly something which is done as a voluntary effort, for free, by Norwegian GPs, I have great faith in*.

(Male GP, Hordaland)

For the GPs to believe in the recommendations, it seemed necessary that they trusted the evidence behind the guidelines. The participants were sceptical about transferring evidence produced in population based studies or hospital based experiments to general practice and individual patients. Many were of the opinion that guidelines developed by hospital specialists had little relevance to general practice and that possible side-effects were often not sufficiently examined. Others had more faith in the specialists' recommendations and were happy to leave some of the responsibility for clinical decisions to those who they assumed had better knowledge of the evidence.

*Yes, we don't really know much about how it is for that particular person. This is aggregated data, and we don't know anything about what the individual's risk level actually is. We can try to work it out, but it is based on statistics*.

(Male GP, Hordaland)

*It's not certain that the gain is as great as we think. They haven't done studies on this population. Guaranteed not. Studies can be done for populations with higher risks than the normal population. There has to be someone sick to be able to measure the difference*.

(Male GP, Hordaland)

*I think you maybe feel a bit of a need to believe (in guidelines). I have to believe some of what comes from the sources we trust. Because there are so many choices, I feel, in this job. And then it's a bit like sharing some of the responsibility in a way*.

(Male GP, Oslo)

A recurring theme in the interviews was how to relate to the fact that recommendations are constantly changing and evidence often debated, reflecting disagreement among the experts. Generally the participants accepted that guidelines are changing and preferred guidelines that were constantly updated, and they saw open discussion as an advantage which increased trust in the guidelines. Hence it was vital that they had had the opportunity to familiarise themselves with the evidence. On the other hand the same participants also sometimes said that they found expert disagreement confusing, especially if they had not had the time to assess the evidence themselves.

Some pointed out that disagreement and debate is positive also because it suggests that it is legitimate to be critical to the recommendations. Others had the opposite reaction; in cases where there is a lot of disagreement about what is best practice, it is safer and easier to stick to the guidelines.

*Well, it's not just one person sitting giving their opinion. It's a discussion and a process the whole time. That gives me more faith in it*.

(Male GP, Oslo)

*GP1: Discussion is an advantage*.

*GP2: Yes, it confirms that we are involved in an academic discipline, not just some kind of slavery*.

*GP1: Yes, that we have to listen to differences and then we have our own experiences. When we have treated 100 patients with blood pressure drugs, we get a bit of a feeling of what gives side-effects and what is effective reducing blood pressure*.

(GP1: Female GP, Oslo; GP2: Male GP, Oslo)

*I'm pretty gullible. I believe everything I hear! (laughs). I believe in a way what is written in the Norwegian medical journal. That I believe. Right until someone writes some weighty critique of those articles which makes me realise, 'Oh, so that wasn't quite right at all then?' So, what I like is maybe places where you see that it's a discussion, the way it is in the journal. It's, in a way, open, a place where you get research and theories presented, but there's a chance for others to come with counter-arguments. And they do that to a large extent. And that makes me, in a way, a bit secure that here you get all kinds of opinions, and if something is completely wrong, then if I read the next journal, I'll realise*.

(Female GP, Oslo)

#### 2. Are the recommendations consistent with this patient's needs?

A recurring theme in the interviews was whether following the guidelines would allow the individual patient's needs and expectations to be fulfilled. The GPs emphasised that they had first hand knowledge of the patients' ailments, expectations of the consultation and life circumstances. People's anxiety about potential health problems and their knowledge about the possibilities for screening and treatment as well as about their rights as patients were frequently discussed in the groups as potential obstacles to adhering to guidelines. Some patients were seen as demanding and several participants emphasised a desire to get patients' appreciation. In the participants' experience, the aim of satisfying patients frequently conflicted with adhering to guidelines. On the other hand a few GPs reported that they referred to guidelines to avoid having sole responsibility for uncomfortable rationing decisions.

*If ***we ***want to survive. If we are going to find a solution for the patient sitting opposite us, we have to compromise on a lot of these guidelines. We have after all learnt to sniff our way forward to what is possible and maybe what is best for the patient. Even if we don't follow all the guidelines*.

(Male GP, Hordaland)

*And patients have their own preferences too. Regarding use of medicines, what symptoms they can tolerate, and what level of blood pressure, what risk they are willing to take, how they are willing to let medication influence their life and what medicines they are prepared to take*.

(Male GP, Hordaland)

The situation where guidelines recommended more treatment than the GPs or the patients themselves deemed suitable was discussed less. In some cases GPs said that they respected and understood patients' wishes to abstain from treatment.

*I had a patient. He was nearly 80 years old and a bit confused, but I had the discussion. Shall we try Maravan? And I put forward the arguments for and against, that you can reduce the risk of brain haemorrhage, but on the other hand, if you start messing about with your medicines, that can be risky too. We agreed after a very pleasant discussion that we would use Albyl instead. It is not so well documented, but it was acceptable, and then you avoid a blood test every four weeks. So I felt that was very meaningful, and the patient lived several more years and died of something completely different*.

(Male GP, Hordaland)

*Guidelines are often very schematic, and life isn't always so simple and so you have patients who are very sceptical about medicines or who have been allergic to loads of stuff, and would rather not have it even if the guidelines say they should. ... There is something about life not being so simple that you can slot it into a framework. You have to have a bit of understanding for that, have a dialogue with the patient, and try to work towards a solution that functions, and sometimes that isn't quite according to the book*.

(Female GP, Hordaland)

#### 3. Is following the recommendations feasible?

The participants reported busy days in their practices. They felt there was no time to update oneself on the medical literature or on the evidence behind the guidelines or perhaps even to read guidelines. During consultations the GPs depended on the guidelines being quick and easy to look up and read. Following the recommendations was often regarded as too time consuming if it involved explaining or convincing patients. Some also noted that it is sometimes difficult to make patients accept a change in guidelines, especially when patients are used to a treatment that is no longer recommended.

*There is a lot of good work behind the formulation of these guidelines. But they become so detailed that I can't remember more than a little. Can always look it up, but you don't have time when you sit there with a patient or you are already a bit behind schedule*.

(Male GP, Hordaland)

It's clear, when you change guidelines, like with these allergy medicines, which came into force on the first of May, in the middle of the worst pollen season, and where specialists have a practice where one medication has been preferred, and most of the patients are on it. And then, first, patients ring to order a repeat prescription, and you have to argue that they should try some other things first, then you have to check if they have used it, you have to test it out, whether it works as well or if there are side effects, and then you have to write an evaluation in the record, and then, if it for one reason or another didn't work properly or had side effects, you can change back again. You find yourself having to argue it out every time you write a new prescription. There is a terrible amount of extra work in that!

(Female GP, Hordaland)

In general participants thought that GPs should be informed about which guidelines to follow and where to find them. They had no time to search the internet for guidelines and their revisions. They found it difficult to find the relevant guidelines since they appear in different sources.

*The regulations for reimbursement of medicines are pretty complicated. It takes a lot of time to understand them. The worst thing is that no-one sends them to you. You just have the responsibility of reading them on the internet and you don't even know what you have to read*.

(Male GP, Hordaland)

*You don't have time after a 50 hour working week to sit in the evenings and read on the internet*.

(Female GP, Hordaland)

Among existing guidelines the GPs preferred those that were easy accessible. The format should be short and simple enough to read in a short time, albeit comprehensive enough to be convincing. Guidelines with access to updated and graded recommendations, depending on the strength of the evidence base, were both easier to trust and easier to "sell" to patients. Electronic sources were generally rated positively for their easy access and convenience when looking up a specific problem or guideline. One advantage of electronic sources was that information could be checked without the patient being aware of it, which made it easier to be convincing. Many mentioned the need for patient leaflets.

In addition, the process of implementation was discussed several times. A transparent process demonstrating the rationale behind the guidelines with a thorough introduction was preferred.

*The good thing about NEL (Electronic Handbook for Norwegian Doctors) is that it gets updated. You know they are written by GPs for GPs. They write up the references. And it is built up in a way that suits the way we work everyday. If we know what it is, wonder if there is anything new in the treatment, we can just click to it and get straight to the chapters. It's easy*.

(Male GP, Hordaland)

*(About guidelines on antenatal care) They have credibility. Partly because they have graded the strength of the advice. Where the documentation is weak, they are cautious. Where the documentation is strong, they are more definite (...) They look systematically at the validity of the research and that also allows us to evaluate each recommendation, how much weight we should put on it, how much we should be inclined to follow it. So that for me is an example of a pretty good guideline. A process which allowed for discussion*.

(Male GP, Hordaland)

*I think most of us have pretty limited time to go systematically through new evidence (...) An easy to use format will have more effect than one which is not so easy to use. Regardless of whether is has a sound basis or is reasonable*.

(Male GP, Hordaland)

## Discussion

The participants were asked to mention and discuss guidelines they knew about and related to in their practices, and this resulted in enthusiastic discussions about a relatively low number of guidelines. The finding that the GPs seem to relate to only a few guidelines does not come as a surprise. Treweek et al's study of reported guideline adherence also found a lack of knowledge about Norwegian guidelines [8], and the best known guidelines are similar in the two studies.

As in earlier studies, the GPs in this study complain about lack of time to update themselves on evidence and the requirements of new guidelines. This discussion was closely linked to scepticism about the evidence. Some of the participants indicated that when they had the time and opportunity to update themselves on the evidence and guidelines and to inform patients, they were far more positive about the guideline recommendations, and found it easier to convince patients. Participants often distrusted governmental guidelines and also repeatedly expressed concern about the influence of the pharmaceutical industry, which they related to the introduction of new drugs or treatment goals (prescriptive guidelines): Several GPs voiced a fear that economic gain motivates undue recommendations and this may lead to medicalisation. The participants agreed that general practitioners should be involved in developing guidelines for general practice. This is in line with earlier studies associating guideline adherence with participation in guideline construction [18].

The reasons for scepticism towards guidelines emerging in this study are comparable to barriers identified in earlier European and US studies including Langley et al's general guideline study and the recent surveys of guideline adherence among Norwegian GPs [3,8,12,19]. This overall picture was expected inasmuch as Carlsen et al's [2007] qualitative review noted similar types of barriers across countries. However, two conspicuous aspects of our results have not been highlighted in earlier studies and merit further discussion; the participants concern with cost issues and their predominantly positive view of changing evidence.

Through open in-depth discussion about attitudes to guidelines in general, the underlying concern that economic motives may be overruling clinical motives emerged as a key finding, and the most obvious reason for distrusting guidelines in this study was GPs' fear that economic considerations were weighted more than clinical considerations. This attitude emerged very clearly through the group discussions even though the participants were not specifically asked about economic considerations. Similar findings have been mentioned in a few earlier studies, but it has not been found to be a central concern [16,20]. A fear of clinical practice guidelines being used as covert tools of rationing may arise, if the purpose and true rationale of the guidelines is not discussed openly [21]. In the Norwegian setting the scepticism seems relevant as several of the guidelines discussed in the interviews are regulatory or include elements of rationing, while there are no sanctions or rewards related to guideline adherence. In contrast, GPs in comparable countries like The Netherlands, Denmark and the UK have been subjected to more extensive demands to practice cost effectiveness; in the UK the new 2004 GP contract employs economic incentives to increase performance and guideline adherence. Recent UK evaluations suggest that the economic incentives are effective with respect to enhancing guideline adherence [22]. Also the debate about using regulatory guidelines seems to have ceased in these countries which could indicate that the GPs' have accepted the need to ration health care.

Regarding proscriptive guidelines, the participants in this study were much concerned about how economic considerations influence the development of guidelines, how economic concerns relate to clinical concerns, whether the authorities can be trusted to divert resources saved to where they can be used more effectively, and how rationing in face to face consultations is a difficult task. In addition the GPs were sceptical about the quality of the cost-effectiveness analyses the guidelines are based on, whether for example the costs of changing established routines are included in the analyses. These concerns are probably well founded, for example, a comprehensive review of guideline implementation by Grimshaw and colleagues [2004] pointed out that costs of the implementation process are rarely included in guideline efficiency studies. Also, empirical evidence that guidelines have contributed to reduced costs is at best limited [23].

The claim that the health services should be cost effective is a well-known issue of conflict between health authorities and health care decision makers [24-26] and the dilemma of balancing cost and care tied to the general practitioners' gatekeeper role have been described in earlier studies [27-29]. US studies of physicians' professional culture indicate that a central ideal is that costs should not be a factor in medical decision making [30]. Likewise Carlsen et al [2007] find that when guidelines appear to be economically motivated (proscriptive guidelines), protection of professional autonomy is given as a reason for not following guidelines. In Norway, the debate about the role of cost containment in health care decisions is constantly newsworthy. The practice of measuring the cost-effectiveness of medical interventions is relatively new, e.g. the decision that new drugs should be subject to a cost-effectiveness analysis before being recommended for reimbursement was only introduced in 2002 [31].

In our interviews the concerns about economic agendas were closely linked to the GPs degree of trust in the authors of the guidelines. Scepticism towards guideline authors has been noted as a barrier in earlier studies. Part of the reason why concerns about economic motives appear in our findings, may be that the general nature of the discussions in this study allowed the participants to explore their own reasoning and motivations. Our study suggests that the underlying reason for distrust in authors and guidelines is the fear that clinical concerns will be jeopardised by economic considerations.

Another theme frequently discussed in the interviews was how changing recommendations and disagreement about evidence influence attitudes. This is the second interesting finding in this study, because it only partly supports Rashidian et al [2007] and other studies [12,14,16,20,32] who found that changes in recommendations evoke negative attitudes. Rashidian et al warn that this change might be a barrier to evidence based practice. Our findings, on the other hand, indicate that although the participants sometimes found changing recommendations confusing, they still welcomed debate about and updates of guidelines. Some of the explanation for this could lie in the Norwegian context which has a relatively small number of guidelines to keep track of. It is also possible that evidence based medicine and transparent academic debate has become more accepted since the other studies were conducted. Sometimes the participants made apparently contradictory statements, stating firmly that they preferred debate and trusted guidelines in spite of changing recommendations, and later in the interview the same participants would describe how changing evidence confused them because they felt unable to update themselves on research. The contradictory statements could suggest that one of the statements represents the politically correct view while the other statement, (arguably the one emerging as the participants become caught up in the discussion), reveals the true feelings about changing guidelines. However, the statements may also reflect true mixed feelings about the inherent uncertainty of medical science, nevertheless combined with a sincere wish to have the opportunity to assess the confusing evidence oneself.

It is also interesting that some of our informants see professional debate and disagreement as an affirmation of their right to use their own independent professional discretion. Paradoxically, if this is the case the GPs may well have positive attitudes towards changing evidence, but the outcome may still be that they choose not to follow guideline recommendations.

### Strengths and limitations

In contrast to the majority of earlier qualitative studies of GPs' views of guidelines, we chose to make the interviews open and allow them to be dominated by the participants, and we did not restrict the discussion to one or more specific guidelines. This enabled us to identify a deeply felt concern about economic motives behind guidelines that has not been reported in detail before. It has been pointed out that a danger with this approach is that it may lead the participants to produce general statements from which it is difficult to draw policy implications [19]. However, we invited the participants to illustrate their points with examples from their practices, and they did so frequently, so their private beliefs were based on experience. Nevertheless, considering readability, only a few elaborate examples could be fitted in to the paper, and most cited statements represent concluding remarks made by participants summing up detailed stories from their practices.

Apart from the fact that only one or two earlier studies have explored GPs' attitudes to guidelines in general, an important reason for choosing a general approach was that we wanted to explore underlying and common problems in GPs' use of clinical recommendations so that this could be of use to health authorities when they are planning strategies of guideline implementation. We were not interested in how a particular guideline was received or could be improved.

While small scale interview studies are unsuitable for surveying the distribution of different attitudes within a group, they are expected to yield data about commonly accepted norms and attitudes [33]. In this study we were aiming at discovering these mainstream attitudes among Norwegian GPs. Thus, if discussion in the groups is dominated by statements that are politically correct among GPs, this is not necessarily problematic. It is to be anticipated that dominant group members influence the statements and opinions of others, as they do in "real" group meetings and other social settings. In this particular case, it seemed to the researcher that the participants felt relaxed within the groups; for example the discussions were easy to spur and practically all participants contributed eagerly and frequently confronted each others opinions. This outspokenness may be ascribed to the participants' familiarity with each other.

Even though our main focus was to reveal general attitudes among the participants, we found that it was sometimes necessary to demonstrate variance in views within the participant group. We have used expressions like "some" or "a few" in contrast to "frequently" and "generally" to denote the difference between consensus and nonconforming voices. This represents a common solution to what we see as a yet unsolved challenge when reporting qualitative findings. Exact counting of statements is normally not useful in semi-structured group interviews because not all participants will answer all questions or make comparable statements about all subthemes, but expressions like "most" or "generally" are used to underline a general and strong tendency in the data. We do not, however, see this as an ideal solution and are welcoming debate as to how qualitative data may be presented in a better way.

We have reflected on the researcher's role in these interviews. One initial concern was that the researcher could be perceived as an agent for the government since the project was funded by the Research Council of Norway which is a governmental institution. The interviewer therefore emphasised her independence as a researcher. The fact that the groups existed as educational groups prior to the research project appeared to inspire confidence and free discussion within the groups with relatively little attention to the researcher's role and background.

The standard caution for in-depth studies is to be aware that the findings are not necessarily valid in other settings. This also applies to this study. Nevertheless, to provide the reader with some idea of the generalisability, we have included a profile of the sample and all Norwegian GPs. We have also provided some of the special context-specific characteristics of Norwegian general practice and the kind of guidelines used. The fact that the overall picture of concerns of the participating GPs is similar to that found in earlier European and US studies as well as in Treweek et al's [2005] Norwegian study, increases the probability that the findings are applicable beyond the context of this study. However, more general studies of attitudes towards guidelines are needed to substantiate our findings. As noted above, the particular concern with economic considerations or the relative lack of concern with changing evidence may be typical for the Norwegian context, but there is no reason to believe that the message this conveys that GPs' scepticism is associated with lack of transparency and involvement in guideline development is specific of the Norwegian context.

A limitation of interview data is that they yield no certain information about the connection between attitudes and actions. Research indicates that positive attitudes to guidelines do not necessarily mean that doctors follow their recommendations [23,34]. A related problem is that it is difficult to separate underlying reasons from what we might call 'excuses'. This can be illustrated by the relationship between the three main barriers depicted in this study; distrust in guidelines, patients' desires and practical problems: When it comes to proscriptive guidelines, doctors have been accused of blaming patients and practical problems for what is actually a lack of motivation for rationing according to the guidelines [35]. On the other hand, there is no doubt a constant lack of time to follow up complicated and impractical guidelines may be a real obstacle to motivation and trust in them. For example, there is evidence that the format of guidelines strongly influences attitudes towards them [36]. Furthermore, patient preferences are supposed to be included in medical decision making, and patient preferences are, as these findings show, influenced by whether or not there is time to give patients reasonable information and whether the GP trusts the evidence. As the three factors have been described several times earlier in comparable studies, it is plausible that they all influence adherence to guidelines and that they all affect each other.

It is also thought provoking that British GPs seem to have put aside the above concerns in favour of economic rewards [22]. If attitudes conveyed in interview studies are so easily influenced by economic incentives, are they then mere excuses for taking the easy way out in practice? Again it may be argued that the fact that private economic concerns are of importance to GPs does not necessarily mean that other concerns are unimportant, especially not in a setting, such as the Norwegian, where the government hitherto has chosen not to employ economic incentives to implement guidelines. Rather, the possible fragility of professional and individual concerns that the UK study gives indications of, and these Norwegian GPs seem to worry about, may be used as an argument against introducing economic incentives.

## Conclusion

The findings suggest that Norwegian GPs should be involved in the development of guidelines, as they already are in some other countries. The evidence also supports the idea that the process of developing guidelines should be transparent; guidelines should be explicit about the underlying purpose, especially when economic considerations are part of the motivation. Comprehensive information about the clinical and economic evaluations of guideline recommendations should be available and this should include calculations of the cost of changing established routines. The recommendations themselves need to be short, simple and include patient leaflets.

## Abbreviations

GP: General Practitioner; NICE: National Institute for Health and Clinical Excellence; COPD: Chronic Obstructive Pulmonary Disease; NEL: Electronic Handbook for Norwegian Doctors.

## Competing interests

The authors declare that they have no competing interests.

## Authors' contributions

BC designed the study. BC and OFN developed the interview guide. BC collected the data. BC and OFN analysed the data. BC drafted the manuscript. BC and OFN revised and approved the manuscript.

## Pre-publication history

The pre-publication history for this paper can be accessed here:


